# A 2-year follow-up to a randomized controlled trial on resistance training in postmenopausal women: vasomotor symptoms, quality of life and cardiovascular risk markers

**DOI:** 10.1186/s12905-024-03351-1

**Published:** 2024-09-13

**Authors:** Sigrid Nilsson, Moa Henriksson, Mats Hammar, Emilia Berin, Sofia Sederholm Lawesson, Liam J. Ward, Wei Li, Anna-Clara Spetz Holm

**Affiliations:** 1 Department of Obstetrics and Gynecology in Linköping, and Department of Biomedical and Clinical Sciences, Linköping University, 58185 Linköping, Sweden; 2 Department of Forensic Genetics and Forensic Toxicology, National Board of Forensic Medicine, Linköping University, Linköping, Sweden; 3 Department of Cardiology, and Department of Health, Medicine and Caring Sciences, Linköping University, Linköping, Sweden

**Keywords:** Cardiovascular risk markers, Menopause, Midlife, Health-related quality of life, Physical activity, Vasomotor symptoms, Hot flushes, Follow-up

## Abstract

**Background:**

Most women experience vasomotor symptoms (VMS) during the menopausal transition. A 15-week resistance training intervention (RTI) significantly reduced moderate-to-severe VMS (MS-VMS) and improved health-related quality of life (HRQoL) and cardiovascular risk markers in postmenopausal women. Whether a short RTI could have long-term effects is unknown. We aimed to investigate whether there were intervention-dependent effects two years after a 15-week RTI on MS-VMS frequency, HRQoL, and cardiovascular risk markers in postmenopausal women.

**Methods:**

This observational prospective cohort study is a follow-up to a randomized controlled trial (RCT) on a 15-week RTI in postmenopausal women (*n* = 57). The control group had unchanged low physical activity during these first 15 weeks. At the follow-up contact two years post-intervention, 35 women agreed to participate in an additional physical visit at the clinic with clinical testing, blood sampling, and magnetic resonance imaging, identical to the protocol at the baseline visit at the start of the RCT.

**Results:**

Although all women showed reduced MS-VMS and increased moderate-to-vigorous physical activity (MVPA) over the 2-year follow-up compared to baseline, the groups from the original RCT (intervention group; IG, control group; CG) changed differently over time (*p* < 0.001 and *p* = 0.006, respectively) regarding MS-VMS. The IG maintained a significantly lower MS-VMS frequency than the CG at the 6-month follow-up. At the 2-year follow-up, there was no significant difference between the original RCT groups. No significant changes over time or differences between groups were found in HRQoL or cardiovascular risk markers. However, significant interactions between original RCT groups and time were found for visceral adipose tissue (*p* = 0.041), ferritin (*p* = 0.045), and testosterone (*p* = 0.010).

**Conclusions:**

A 15-week resistance training intervention reduced MS-VMS frequency up to six months post-intervention compared to a CG, but the effect was not maintained after two years. The RTI did neither contribute to preserved improvements of cardiovascular risk markers nor improved HRQoL after two years compared to a CG.

**Trial registration:**

Clinical trials.gov registered ID: NCT01987778, trial registration date 2013–11-19.

**Supplementary Information:**

The online version contains supplementary material available at 10.1186/s12905-024-03351-1.

## Introduction

Hot flushes and night sweats (i.e., vasomotor symptoms, VMS) are the most common menopausal symptoms, affecting up to 80% of women during the menopausal transition and accompanying the decrease in estradiol due to ovarian failure [1]. The VMS may impair sleep, cognition, mood, and quality of life [2]. Menopausal hormone therapy (MHT) is the most effective VMS treatment. In a randomized controlled trial (RCT), we found that a 15-week resistance training intervention (RTI) reduced moderate-to-severe VMS (MS-VMS) frequency by almost 50% in the intervention group (IG) compared to the control group (CG) with unchanged physical activity [3]. Additionally, health-related quality of life (HRQoL) was improved in the IG [4].

Some, but not all [5], observational data suggest that experiencing VMS is independently associated with subclinical [6] and established cardiovascular disease (CVD) [7, 8]. Women of fertile age are partly protected against CVD, as estrogen counteracts the early atherosclerosis process [9]. After menopause, women develop a more atherogenic blood lipid profile, systemic inflammation [10], as well as visceral fat redistribution, which are considered cardiovascular risk markers [11]. The CVD risk enhancement associated with the menopausal transition can be reduced with physical activity (PA) [12]. We found that a 15-week RTI was beneficial in treating VMS in postmenopausal women [3] and reduced atherogenic blood lipids, ferritin, biomarkers of inflammation, and adipokines short-term [13, 14].

The current study aimed to investigate the effects of a 15-week RTI on MS-VMS frequency and HRQoL in postmenopausal women six months and two years post-intervention. We also aimed to evaluate whether the RTI could improve the natural menopause- and age-dependent cardiovascular risk marker deterioration and redistribution of adipose tissue. The main hypothesis was that a structured RTI could improve MS-VMS frequency and HRQoL over time compared with a group that received a less structured introduction to resistance training after the initial 15 weeks of unchanged low PA (herein referred to as the control group, CG). We also hypothesized that an RTI may contribute to improved cardiovascular risk markers over time for postmenopausal women with VMS compared with a CG. The above hypotheses are possibly explained by an RTI-induced production of hypothalamic opioids, thereby lowering the thermoregulatory instability facilitating VMS activation, but also by reducing low-grade inflammation, improving endothelial function, and body composition.

## Methods

### Primary and secondary outcomes

The primary outcome was the intervention-dependent change in MS-VMS frequency and HRQoL over time. The secondary outcomes were the intervention-dependent changes over time in cardiovascular risk markers (i.e., blood pressure (BP), body anthropometry, blood lipids, ferritin, biomarkers of inflammation, adipokines, sex hormones, and abdominal fat assessed with magnetic resonance imaging (MRI)).

### Study design and population

#### Study design

The present observational prospective cohort study is a follow-up to an open, parallel-group RCT of the effect of a 15-week RTI on VMS performed at the Department of Obstetrics and Gynaecology of the University Hospital in Linköping, Sweden. The study protocol has been preregistered (clinicaltrials.gov ID: NCT01987778) and published [15], and adheres to STROBE guidelines.

The RCT included two post-intervention telephone follow-ups, at six months and two years after the intervention, during which information was retrieved about general health, change of medication, and physical activity (PA). Two weeks before the telephone follow-ups, VMS diaries and questionnaires about HRQoL and PA were sent to the participants by post. The telephone interviewer did not have the diaries and questionnaires present during the follow-up call. After the 2-year telephone follow-up, 35 women agreed to attend additional data collection at the outpatient clinic, which included measuring BP, body anthropometry, blood sampling, and MRI (i.e., the same measurements as at the baseline visit at the start of RCT). The dataset on VMS, HRQoL, and cardiovascular risk markers from the baseline and week-15 visit has been previously published [3, 4, 13, 14]. The period for the additional visit was set to 24–28 months post-intervention/control period to enable more participants to participate.

#### Population

**Fig. 1:**
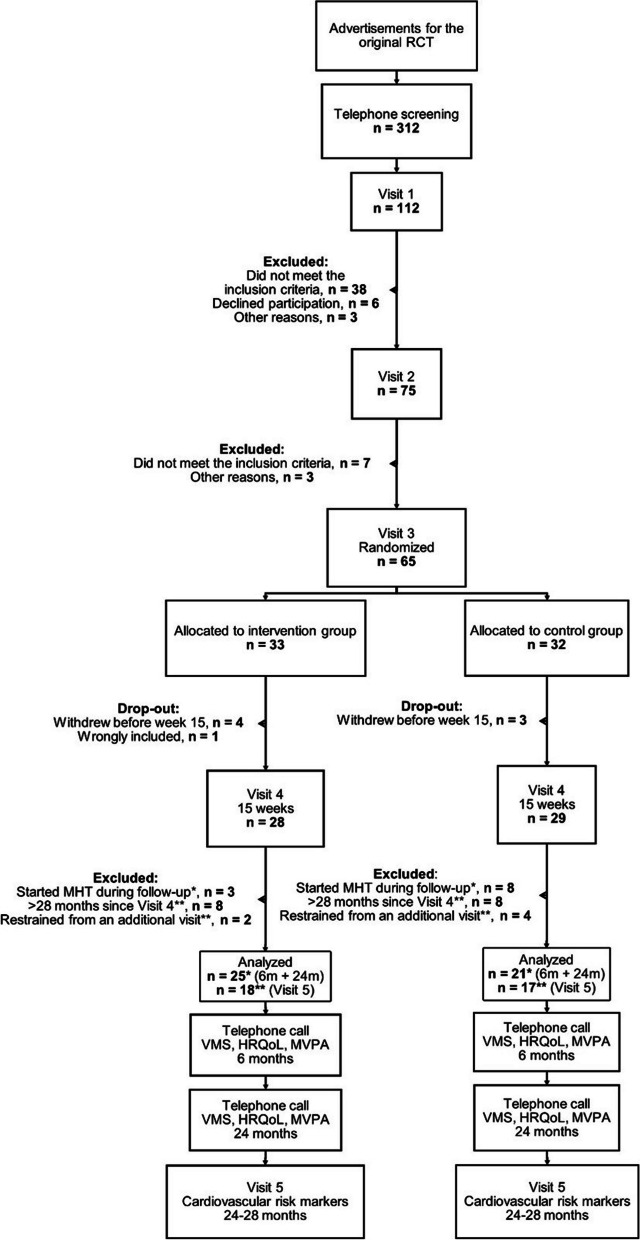
Flowchart. * Only relevant for the VMS and HRQoL outcomes. ** Only relevant for the cardiovascular risk marker outcomes. RCT, randomized controlled trial; MRI, magnetic resonance imaging; MHT, menopausal hormone therapy; VMS, vasomotor symptoms; HRQoL, health-related quality of life; MVPA, moderate to vigorous physical activity

The number of participants at baseline, 15 weeks, and each follow-up is shown in Fig. 1. In total, 65 postmenopausal women with low PA were included and randomized to either 15 weeks of RTI (IG) or a control group (CG) with unchanged low PA. Low physical activity (PA) was defined as ≤ 225 min per week of mixed physical intensity and/or ≤ 75 min per week of moderate-to-high intensity PA, i.e., it feels strenuous and makes you breathe heavier than usual (e.g., brisk walking, Nordic walking, cycling, aerobics, running, or any other form of exercise). At 15 weeks, 57 participants remained: 28 in the IG and 29 in the CG. Initiation of MHT during follow-up was an exclusion criterion for the VMS and HRQoL outcomes, excluding three women in the IG and eight women from the CG. The remaining 46 participants (25 in the IG and 21 in the CG) constituted the study population for these primary outcomes during the 6-month and 2-year follow-up. Exceeding 28 months since the 15-week visit (Visit 4) or unwillingness to attend an additional visit to the clinic were exclusion criteria for the secondary outcomes, which included body anthropometry, blood sampling, and MRI (i.e., cardiovascular risk markers). After evaluating the eligibility criteria for the secondary outcomes, 18 in the IG and 17 in the CG attended the follow-up after 24–28 months (Visit 5). These participants constituted the population for the secondary outcomes (Fig. 1).

### Study procedures

RCT participants (*n* = 65) were block randomized in a 1:1 allocation by an independent statistician to RT or unchanged low PA for 15 weeks. The participants were non-blinded to the intervention. Sealed sequentially numbered envelopes were opened by each woman in the presence of the gynecologist. A statistician not belonging to the research group performed the randomization process and order. Investigators were blinded for group allocation until analyses of data had been completed. The whole body RTI included six seated machine resistance-training exercises and two body-weight exercises, performed with 8–12 repetitions in 2 sets [15]. The same physiotherapist constructed and supervised the RTI, throughout the study period [16]. Intervention compliance was defined as an average attendance to ≥ 2 weekly sessions, controlled by gym electronic card system logs. Training sessions were supervised by the physiotherapist at least once weekly. The CG was asked to maintain their low baseline PA level during the 15-week study period.

After 15 weeks, the CG was offered a four-month gym membership and one physiotherapist-led resistance training introduction. E-mails were sent to all participants in the IG and CG twice yearly during follow-up, providing information about the general health benefits of PA and preliminary RCT results.

### Measures

#### Vasomotor symptoms (VMS)

During the two consecutive weeks before the 6-month and 2-year follow-ups, participants registered daily mild, moderate, and severe VMS, PA amount, health complaints, and medications in diaries. VMS intensity was defined as follows: 1) mild: VMS limited to a heat sensation without sweating; 2) moderate: VMS experienced as both a heat sensation and sweating, but not to the extent that it hinders ongoing activity; 3) severe: VMS experienced as a heat sensation and sweating that forces the woman to stop ongoing activity or causes awakening during sleep.

VMS frequency per 24 h was calculated as the sum of VMS divided by the reported number of days. MS-VMS was calculated as the sum of the mean MS-VMS frequencies. Hot flush score (HFS) was calculated by multiplying the mean frequencies of mild, moderate, and severe VMS by one, two, and three, respectively. The sum of each severity domain product per 24 h generated the HFS per 24 h.

#### Health-related quality of life (HRQoL)

To assess menopause-specific HRQoL, the Swedish version of the Women’s Health Questionnaire (WHQ) was used [17], which has shown good validity in postmenopausal women [18]. The WHQ includes 36 items answered on a 4-point scale, generating scores that result in nine domains: depressed mood, somatic symptoms, anxiety, VMS, sleep problems, sexual behavior, menstrual symptoms, memory/concentration, and attractiveness [17, 19]. Each symptom domain score ranges from 0 to 1, where a higher score corresponds to a greater symptom burden.

Generic HRQoL was evaluated with the Swedish version of the Short-Form Health Survey (SF-36), which includes 36 items to assess eight health dimensions: physical functioning, role limitations due to physical problems, bodily pain, general health perception, vitality, social functioning, role limitations due to emotional problems, and mental health. For each dimension of health, a score ranging from 0 to 100 is calculated, and a higher score corresponds to better functionality. Two summary scores are also generated from the scores in each health dimension to assess the physical and mental components separately.

#### Basic clinical information and anthropometric measurements

Information about age, menopausal time, medication use, disease history, and smoking were collected at inclusion in the original RCT. BP and anthropometry were collected at baseline, week 15, and the follow-up visit 24–28 months after the initial 15 weeks (Visit 5).

#### Biochemical assays

Fasting venous blood samples were drawn and collected in EDTA vacutainers (BD AB, Sweden) at baseline, week 15, and 24–28 months after the initial 15 weeks (Visit 5). Hemoglobin, iron status, and lipid profile were analyzed consecutively as described [13] according to clinical routine in an accredited laboratory (ISO/IEC 17025) at the Department of Clinical Chemistry, Linköping, Sweden. Due to logistical reasons, testosterone, sex hormone-binding globulin (SHBG), and high-sensitive C-reactive Protein (hsCRP) were analyzed by the same accredited laboratory in one batch but after storage in freezers and thawing.

Plasma biomarkers were analyzed as previously described [14], according to the manufacturer’s guidelines, using the immunoassay MILLIPLEX (Merck, Germany) with xMAP technology (Luminex Corporation, TX, USA). Analyses were spread across 11 bead panels, measuring 37 analytes, of which 14 were within detectable limits. These analytes included adiponectin, adipsin, brain-derived neurotrophic factor (BDNF), interleukin-1 receptor antagonist (IL1-RA), IL-6, IL-10, leptin, lipocalin-2, matrix metalloproteinase 2 (MMP2), MMP9, monocyte chemoattractant protein 1 (MCP1), plasminogen activator inhibitor 1 (PAI-1), resistin, and tumor necrosis factor (TNF-α).

#### MRI-based body composition analysis

MRI images were acquired using a Philip Ingenia 3.0 T MR-scanner (Philips, Best, The Netherlands) and described in more detail elsewhere [20, 21]. The acquisition, reconstruction, fat-referenced image calibration, and image segmentation were previously described [20]. The following body composition measurements were derived from the processed MR images: Visceral Adipose Tissue (VAT); Abdominal Subcutaneous Adipose Tissue (ASAT); Total Abdominal Adipose Tissue (TAAT, i.e. the sum of ASAT and VAT); VAT-ratio (the ratio between VAT and TAAT.

#### Physical activity (PA)

The validated short-form International Physical Activity Questionnaire (IPAQ) was used to evaluate the self-reported PA during follow-up [22]. This questionnaire contains nine questions regarding time (duration and frequency) spent on vigorous and moderate activities, walking, and sitting during the last seven days. MVPA is minutes spent on moderate and vigorous physical activities during the previous week.

### Data analyses

#### Sample size calculation

A sample size calculation was performed on the main outcome measure in the original RCT [15]. Forty women had to be randomized to get 80% power to show a significant difference (*p* < 0.05, two-sided statistical test) in the reduction of VMS between the intervention and control group, anticipating a dropout rate of 20%. To enhance the statistical power of secondary outcomes, we aimed to include 60 participants.

#### Statistical analyses

The intention-to-treat (ITT) principle was applied during analysis for both the primary and secondary outcomes, i.e., all participants who did not actively withdraw their informed consent or fulfilled the exclusion criteria were included according to their original allocation group.

Data distribution determined whether the presented data were expressed as mean ± standard deviation (SD) or median (25th and 75th percentile). Independent statistical analyses between the groups at baseline were performed using the Student’s *t*-test or Mann–Whitney *U* test. All available data at each follow-up visit were included in the statistical analyses.

Linear mixed models (LMMs) with diagonal covariance structure were used to test the primary and secondary outcomes. A priori distribution evaluation of all outcome variables was performed to confirm that the normality assumption criterion was fulfilled. All biomarkers and adipokines were logarithmically transformed before analysis to achieve that criterion. IL-6 and BDNF also had a constant added before logarithmic transformation to avoid negative values.

The main effects investigated as fixed factors were group (IG versus CG), time (treated ordinally with four levels for the primary outcome and three levels for the secondary outcomes), and group x time interaction. Restricted maximum likelihood (REML) was used to achieve more unbiased variance estimates. Reference categories in all analyses were the baseline visit and the CG. For statistically significant variables in the main effects analyses, post-hoc analyses with Bonferroni correction were performed for both the IG and CG to compare the differences across the visits. Sensitivity analyses were performed for the secondary outcomes to explore the effect of potentially confounding variables, e.g., the start of MHT during the follow-up period.

The WHQ variables had skewed distributions that did not fit the LMMs. Therefore, the non-parametric Mann–Whitney U test was used to compare the differences across visits between the original allocation groups. Missing data frequencies and percentages for all variables and outcomes are presented in the supplementary file and not imputed. Results with *p* < 0.05 were considered statistically significant. The analyses were performed using SPSS v.28.0 (IBM, Portsmouth, UK).

## Results

### Vasomotor symptoms (VMS) and health-related quality of life (HRQoL)

Participants were 56 ± 5 years at baseline (Visit 3), with a median postmenopausal time of 41 (17/94) and 46 (21/78) months in the IG and CG, respectively. More detailed characteristics of the randomized participants at baseline have been described elsewhere [3]. The VMS and HRQoL characteristics from each visit and telephone follow-up are presented in Tables 1, 2 and 3. The VMS and HRQoL characteristics did not differ significantly between the groups at baseline (Tables 1, 2 and 3).

**Table 1:** Vasomotor symptoms (VMS)

*Variables*	**IG (*n* = 25)**			**CG (*n* = 21)**			Type III fixed effects^a^		
**VMS/24 h**^§,b^	**BL** ***n* = 25**	**6 m** ***n* = 20**	**2y** ***n* = 21**	**BL** ***n* = 21**	**6 m** ***n* = 19**	**2y** ***n* = 20**	**Group** **F (*p*-value)**	**Time** **F(*p*-value)**	**Group x Time F(*p*-value)**
Moderate	3.0 (2.4/4.2)	1.4 (0.7/2.3)	0.9 (0.1/2.8)	2.9 (1.9/3.8)	2.1 (1.4/2.9)	1.8 (0.9/2.6)	1.627 (0.208)	11.437 (< 0.001**)	4.180 (0.009**)
Severe	3.5 (2.2/6.4)	1.1 (0.3/2.9)	0.1 (0.0/2.1)	2.8 (1.8/4.3)	2.4 (1.1/3.9)	1.6 (0.3/2.4)	1.924 (0.172)	7.218 (< 0.001**)	3.668 (0.018*)
Moderate + Severe	6.1 (4.9/9.1)	2.8 (1.3/5.6)	1.3 (0.3/5.9)	5.7 (4.7/7.8)	4.8 (2.5/6.0)	3.5 (2.0/5.9)	3.641 (0.060)	27.482 (< 0.001**)	9.213 (< 0.001**)
Hot flush score^c^	17.1 (12.9/26.9)	7.5 (4.0/14.9)	3.3 (1.3/14.8)	15.6 (12.0/21.4)	12.7 (7.6/17.1)	9.4 (5.6/15.3)	6.066 (0.016*)	13.944 (< 0.001**)	4.416 (0.007**)

*IG* Intervention group ((i.e., performing a structured resistance training intervention), *CG* Control group (i.e., receiving a less structured introduction to resistance training after the initial 15 weeks of unchanged low physical activity), *BL* Baseline, *6 m* Six months, *2y* Two years

^§^Median (25th/75th percentile); *p < 0.05; ** *p* < 0.01

^a^Linear mixed model (LMMs)

^b^Logaritmized values in the statistical analyses

^c^Hot flush score was calculated by multiplying the mean mild, moderate, and severe VMS frequency by one, two, and three, respectively. The sum of each severity domain product per 24 h generated the HFS per 24 h

**Table 2:** Health-related quality of life (HRQoL), SF-36

*Variables*	**IG (*n* = 25)**			**CG (*n* = 21)**			Type III fixed effects^a^		
**SF-36**^~^	**BL** ***n* = 25**	**6 m** ***n* = 22**	**2y** ***n* = 23**	**BL** ***n* = 21**	**6 m** ***n* = 20**	**2y** ***n* = 20**	**Group** **F (*p*-value)**	**Time** **F (*p*-value)**	**Group x Time** **F (*p*-value)**
PCS	49.8 ± 6.3^¤^	51.1 ± 5.6^∞∞∞^	49.6 ± 8.1^#^	50.4 ± 7.2	48.6 ± 11.7	49.1 ± 9.5^###^	0.454 (0.504)	0.242 (0.866)	1.215 (0.313)
MCS	49.9 ± 9.2^¤^	52.2 ± 6.7^∞∞∞^	49.9 ± 9.6^#^	48.9 ± 10.7	51.2 ± 11.8	51.7 ± 8.4^###^	0.012 (0.912)	2.275 (0.092)	0.924 (0.437)
Physical function	92.2 ± 7.2	96.9 ± 7.2	92.0 ± 8.4	87.6 ± 13.7	86.8 ± 17.3	82.0 ± 24.4	5.323 (0.025*)	1.139 (0.340)	0.717 (0.546)
Role physical	79.0 ± 35.6 ±	88.6 ± 29.6	83.7 ± 29.8	88.1 ± 18.7	82.5 ± 31.5	85.0 ± 28.6	0.030 (0.864)	0.100 (0.959)	0.637 (0.594)
Bodily pain	73.2 ± 21.0^¤^	78.6 ± 18.9^∞∞∞^	74.0 ± 26.1	78.8 ± 21.6	73.6 ± 27.3	74.7 ± 27.5	0.003 (0.955)	0.039 (0.990)	1.042 (0.381)
General health	77.2 ± 14.5¤	78.3 ± 14.5^∞∞∞^	71.5 ± 19.7^#^	76.3 ± 21.1	74.2 ± 21.5	72.3 ± 24.6	0.150 (0.700)	6.835 (< 0.001**)	0.962 (0.417)
Vitality	67.0 ± 18.0^**¤**^	70.7 ± 18.3^**∞∞∞**^	66.8 ± 21.2	61.2 ± 25.1	66.8 ± 24.0	63.5 ± 19.1	0.850 (0.361)	4.489 (0.008**)	1.766 (0.167)
Social function	86.6 ± 18.3^**¤**^	92.9 ± 14.0^**∞∞∞**^	91.4 ± 15.7	87.0 ± 17.9	92.5 ± 16.0	89.4 ± 17.4	0.062 (0.805)	1.460 (0.237)	0.402 (0.752)
Role emotional	90.7 ± 28.1^¤^	97.0 ± 9.7	83.1 ± 30.4	85.7 ± 29.0	83.4 ± 33.3	93.0 ± 17.9^###^	0.033 (0.856)	1.058 (0.376)	2.464 (0.073)
Mental health	81.1 ± 14.1^**¤**^	84.4 ± 11.8^**∞∞∞**^	82.0 ± 15.7	82.4 ± 17.4	85.4 ± 16.6	82.4 ± 18.0	0.141 (0.709)	1.391 (0.259)	0.161 (0.922)

*IG* Intervention group ((i.e., performing a structured resistance training intervention), *CG* Control group (i.e., receiving a less structured introduction to resistance training after the initial 15 weeks of unchanged low physical activity), *BL* Baseline, *6 m* Six months, *2y* Two years, *SF-36* Short Form Health Survey, *PCS* Physical component summary, *MCS* mental component summary; ~ Mean ± SD

^*^p < 0.05; ** *p* < 0.01; ¤ *n* = 24; # *n* = 22; ### *n* = 19; ∞∞∞ *n* = 21

^a^Linear mixed models (LMMs)

**Table 3:** Health-related quality of life (HRQoL), WHQ

*Variables*	**IG (*n* = 25)**			**CG (*n* = 21)**			Mann–Whitney U test	
**WHQ**^§^	**BL** ***n* = 25**	**6 m** ***n* = 22**	**2y** ***n* = 24**	**BL** ***n* = 21**	**6 m** ***n* = 20**	**2y** ***n* = 20**	**Δ15w-6m**^**a**^	**Δ6m-24m**^**a**^
Depressed mood	0.00 (0.00/0.14)	0.00 (0.00/0.04)	0.00 (0.00/0.14)	0.00 (0.00/0.14)	0.00 (0.00/0.14)	0.00 (0.00/0.14)	0.283	0.466
Somatic symptoms	0.14 (0.00/0.36)	0.14 (0.00/0.29)	0.14 (0.00/0.25)	0.14 (0.00/0.29)	0.07 (0.00/0.39)	0.07 (0.00/0.39)	0.562	0.340
Memory/Concentration	0.00 (0.00/0.33)	0.00 (0.00/0.33)	0.00 (0.00/0.33)	0.00 (0.00/0.00)	0.00 (0.00/0.33)^###^	0.00 (0.00/0.33)	0.548	0.936
Vasomotor symptoms	1.00 (1.00/1.00)	0.75 (0.00/1.00)	0.50 (0.00/1.00)	1.00 (1.00/1.00)	1.00 (0.63/1.00)	0.75 (0.00/1.00)	0.867	0.126
Anxiety/Fears	0.00 (0.00/0.00)	0.00 (0.00/0.00)	0.00 (0.00/0.00)	0.00 (0.00/0.00)	0.00 (0.00/0.00)	0.00 (0.00/0.00)	0.306	0.254
Sexual behaviour	0.33 (0.00/0.67)^**¤¤¤**^	0.33 (0.00/0.67)^**##**^	0.33 (0.00 /0.67)^**###**^	0.33 (0.00/0.67)^**∞**^	0.00 (0.00/0.67)^**¤¤**^	0.00 (0.00/0.67)^**∞∞**^	0.305	0.762
Sleep problems	0.33 (0.00/0.67)	0.33 (0.00/0.42)	0.33 (0.00/0.33)	0.33 (0.00/0.33)	0.33 (0.00/0.33)	0.33 (0.00/0.33)	0.283	0.808
Menstrual problems	0.00 (0.00/0.25)	0.00 (0.00/0.25)	0.00 (0.00/0.25)	0.00 (0.00/0.25)^###^	0.00 (0.00/0.25)^###^	0.00 (0.00/0.33)	0.097	0.542
Attractiveness	0.50 (0.25/1.00)	0.25 (0.00/0.50)	0.5 (0.00/0.50)	0.50 (0.50/0.50)	0.50 (0.00/0.50)	0.50 (0.00/1.00)	0.814	0.863

*IG* Intervention group ((i.e., performing a structured resistance training intervention), *CG* Control group (i.e., receiving a less structured introduction to resistance training after the initial 15 weeks of unchanged low physical activity), *BL* Baseline, *6 m* Six months, *2y* Two years, *WHQ* Women’s Health Questionnaire

^§^Median (25th / 75th percentile); ¤¤ *n* = 15; ¤¤¤ *n* = 23; ## *n* = 18; ### *n* = 19; ∞ *n* = 17; ∞∞ *n* = 14

^a^Mann-Whitney U test

**Fig. 2:**
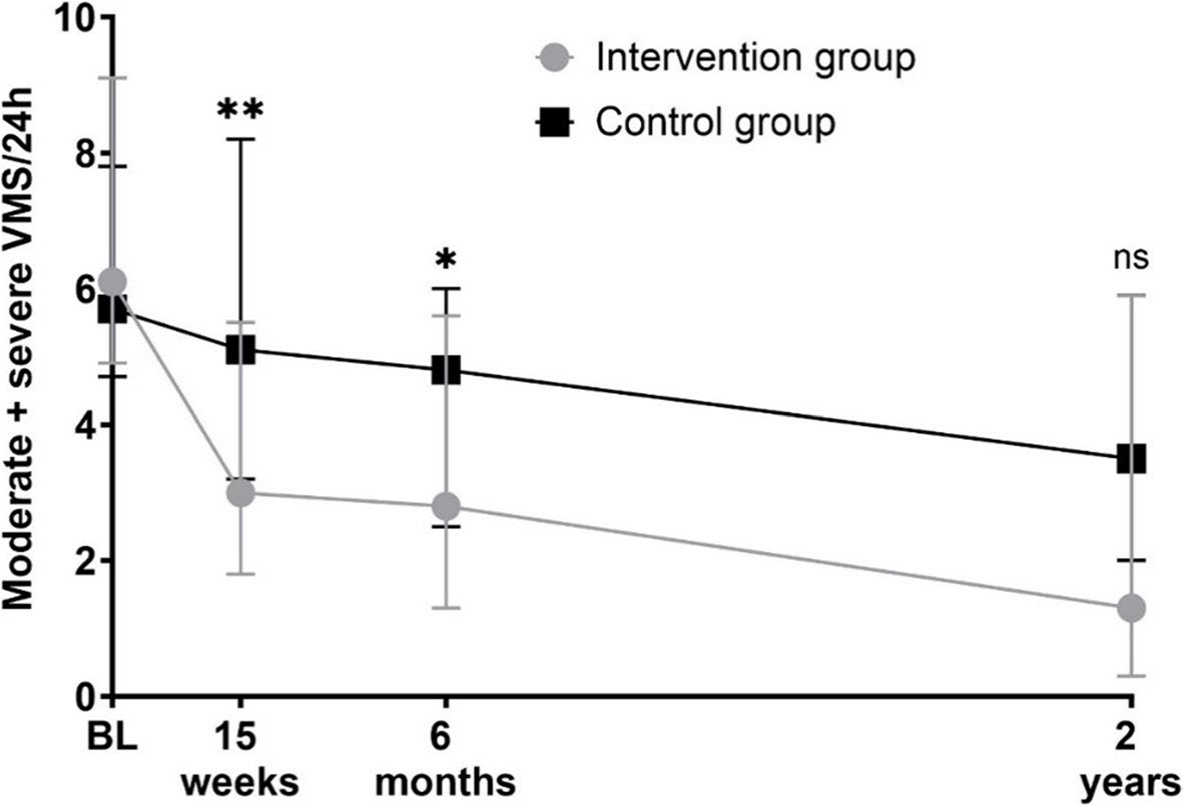
Moderate to severe vasomotor symptoms (MS-VMS) frequency across time. Moderate to severe vasomotor symptoms (MS-VMS) per 24-h period for the intervention group (i.e., performing a structured resistance training intervention) and control group (i.e., receiving a less structured introduction to resistance training after the initial 15 weeks of unchanged low physical activity). Data is expressed at all visits and telephone follow-ups in the RCT. The central tendency and dispersion are expressed as median and 25th/75th percentiles. Post-hoc between-group comparisons at each visit/telephone follow-up are performed with Bonferroni correction. * *p* < 0.01; ** *p* < 0.05; BL, baseline; ns, non significant

The MS-VMS frequency and HFS over time are illustrated in Fig. 2. Significant differences were observed in the MS-VMS frequency and the HFS change over time between the IG and CG, supported by a significant interaction between group and time (*p* < 0.001 and *p* = 0.007, respectively) (Table 1). All women showed a reduction in MS-VMS frequency and HFS over time from baseline in the RCT (*p* < 0.001) (Table 1). There was a group-specific effect on HFS (*p* = 0.016), indicating significant differences between the groups across the visits in the HFS variable. At the 6-month follow-up, both the IG and CG had decreased in MS-VMS frequency and HFS compared to week 15. There was still a significant difference in the post hoc comparisons between groups at six months (*p* = 0.025), with lower MS-VMS frequency in the IG (2.8 HF/24 h) compared to the CG (4.8 HF/24 h). The 2-year post hoc comparisons between groups were non-significant for MS-VMS frequency and HFS, although the point estimates still differed between the groups.

The WHQ variables did not differ significantly between week 15 and the follow-up contacts (Table 3). The general health (GH) and vitality (VT) domains of SF-36 decreased significantly over time for all women, regardless of group (*p* < 0.001 and *p* = 0.008, respectively) (Table 2). There were no significant changes in the SF-36 HRQoL variables between the IG and CG over time. However, a group-specific effect was observed in the physical function dimension of SF-36 (*p* = 0.025) (Table 2), indicating that the intervention group consistently scored higher than the control group throughout the study in this variable.

### Cardiovascular risk markers

There were no significant differences in BP, anthropometry, MRI-assessed adipose tissue, blood lipids, ferritin, and sex hormones between groups at baseline (Tables 4 and 5). Among the biomarkers, lipocalin-2 and PAI-1 were significantly higher in CG at baseline (*p* < 0.001 and *p* = 0.019) (Table 5). All women increased in waist circumference (*p* = 0.003) (Table 4) and decreased in SHBG (*p* = 0.006) (Table 4) and MMP9 (*p* = 0.016) (Table 5) levels over time. The IG had significantly lower levels of IL1-RA across the visits compared with the CG (*p* = 0.025) (Table 5).

**Table 4:** Cardiovascular risk markers

*Variables*	**IG (*n* = 18)**		**CG (*n* = 17)**		Type III fixed effects^a^				
**Clinical information**^~^	**BL** ***n* = 18**	**24-28 m** ***n* = 18**	**BL** ***n* = 17**	**24-28 m** ***n* = 16**	**Group** **F (*p*-value)**		**Time** **F (*p*-value)**		**Group x Time** **F (*p*-value)**
SBP (mmHg)	133 ± 16	130 ± 20	130 ± 16	133 ± 24	0.089 (0.767)		0.377 (0.689)		1.678 (0.201)
DBP (mmHg)	79 ± 7	75 ± 10	79 ± 10	75 ± 11	0.029 (0.867)		3.110 (0.056)		0.412 (0.665)
Weight (kg)	76.7 ± 13.3	75.1 ± 14.4	73.3 ± 11.1	71.9 ± 11.3	0.412 (0.525)		0.590 (0.560)		0.624 (0.542)
BMI (kg/m^2^)	28.2 ± 4.2	27.6 ± 4.8	27.0 ± 3.7	26.5 ± 3.9	0.496 (0.486)		0.921 (0.408)		0.670 (0.519)
WC (cm)	92.4 ± 13.7	97.6 ± 12.1	87.9 ± 13.2	90.7 ± 11.9^¤^	1.035 (0.317)		6.726 (0.003**)		0.603 (0.552)
HAW (cm)	18.5 ± 2.6	18.3 ± 1.9	18.0 ± 2.7	17.7 ± 1.8	0.183 (0.672)		0.171 (0.844)		0.244 (0.785)
SAD (cm)	22.1 ± 3.0	21.9 ± 2.4	21.4 ± 2.8	20.9 ± 2.8	0.257 (0.615)		2.714 (0.078)		2.498 (0.094)
**MRI**^§,^^b,c^	***n* = 15**	***n* = 9**	***n* = 13**	***n* = 9**					
ASAT (L)	8.4 (7.4/12.0)	9.8 (8.3/13.7)	8.1 (6.5/11.6)	9.3 (7.3/11.6)	2.859 (0.097)		0.238 (0.789)		0.068 (0.934)
VAT (L)	3.2 (1.8/3.7)	3.6 (2.5/4.5)	2.5 (1.3/3.6)^#^	2.8 (1.8/3.7)	1.396 (0.248)		2.270 (0.132)		3.836 (0.041*)
VAT-ratio (%)	23.9 (17.3/28.1)	24.6 (19.9/28.8)	22.9 (18.5/25.3^)#^	23.4 (20.5/25.5)	0.777 (0.386)		2.885 (0.082)		1.351 (0.285)
***Blood analyses***	***n* = 17**	***n* = 18**	***n* = 18**	***n* = 16**					
LDL (mmol/L) ^~^	3.58 ± 1.17	3.12 ± 0.69	3.68 ± 0.70	3.60 ± 0.60	1.193 (0.287)	2.320 (0.112)		1.071 (0.353)	
HDL (mmol/L) ^~,c^	2.01 ± 0.65	1.88 ± 0.35	2.18 ± 0.87	1.86 ± 0.50	0.432 (0.513)	1.238 (0.298)		0.244 (0.785)	
ApoA1 (g/L) ^~^	1.82 ± 0.23	1.81 ± 0.21	1.77 ± 0.29	1.78 ± 0.33	0.108 (0.744)	0.008 (0.992)		0.239 (0.788)	
ApoB (g/L) ^~^	1.13 ± 0.33	1.00 ± 0.20	1.11 ± 0.17^¤¤^	1.11 ± 0.19	0.551 (0.463)	2.654 (0.083)		2.233 (0.121)	
Ferritin (μg/L) ^§,b^	100 (51/141)	85 (61/108)	152 (64/169)	145 (76/160)	2.254 (0.142)	2.683 (0.082)		3.398 (0.045*)	
hsCRP (µg/mL) ^§,b^	1.26 (1.03/2.70)	1.29 (0.70/2.09)	1.70 (0.73/2.56)	2.82 (0.50/3.30)	0.198 (0.659)	0.239 (0.789)		0.489 (0.617)	
Testosterone (nmol/L) ^§,b^	0.44 (0.26/0.54)	0.53(0.32/0.73)	0.54(0.21/1.00)	0.67(0.11/0.85)	0.021 (0.855)	0.557 (0.578)		5.351 (0.010*)	
SHBG (nM) ^~^	31.4 ± 14.3	29.0 ± 12.4	33.5 ± 15.8	32.4 ± 17.7	0.141 (0.710)	5.944 (0.006**)		0.936 (0.402)	

*IG* Intervention group (i.e., performing a structured resistance training intervention), *CG* Control group (i.e., receiving a less structured introduction to resistance training after the initial 15 weeks of unchanged low physical activity), *BL* Baseline, *6 m* Six months, 24-28 m, the period within the additional follow-up visit was set post-intervention/control period to enable more participants to participate, *SBP* Systolic blood pressure, *DBP* Diastolic blood pressure, *BMI* Body Mass Index, *WC* Waist circumference, *HAW* Half abdominal width, *SAD* Sagittal abdominal diameter, *ASAT* Abdominal subcutaneous adipose tissue, *VAT* Visceral adipose tissue, *VAT-ratio* Ratio in percent to total body adipose tissue, *LDL* Low-dense lipoprotein, *HDL* High-dense lipoprotein, *ApoA1* Apolipoprotein A1, *ApoB* Apolipoprotein B, *hsCRP* High sensitive C-reactive protein, *SHBG* Sex Hormone Binding Globulin

^¤^*n* = 169; ^~^ Mean ± SD; ^§^ Median (25th / 75th percentile); * *p* < 0.05; ^¤^*n* = 15; ^¤¤^*n* = 16; ^¤¤¤^*n* = 17; ^#^*n* = 12

^a^Linear Mixed Models (LMMs)

^b^Logaritmized values in the statistical analyses

^c^Linear mixed models did not include random effects due to statistical reasons

**Table 5:** Biomarkers and adipokines

*Variables*	**IG (*n* = 18)**		**CG (*n* = 17)**		**Type III fixed effects**^a^		
**Biomarkers & adipokines**^§,b^	**BL** ***n* = 18**	**24-28 m** ***n* = 18**	**BL** ***n* = 17**	**24-28 m** ***n* = 15**	**Group** **F(*p*-value)**	**Time** **F(*p*-value)**	**Group x Time** **F(*p*-value)**
Leptin (ng/mL)	31.5 (18.1/61.1)	36.9 (21.8/55.1)	41.4 (16.5/62.7)	33.5 (18.4/66.4)	0.176 (0.677)	0.582 (0.564)	0.536 (0.590)
MMP2 (ng/mL)	143.7 (126.3/158.8)	138.9 (114.0/149.3)	129.4 (114.5/166.2)	140.6 (119.7/156.9)	0.070 (0.794)	0.583 (0.563)	1.169 (0.320)
MMP9 (ng/mL)	35.7 (24.9/50.0)	32.8 (27.6/41.3)	41.1 (34.4/60.0)	35.9 (27.7/48.3)	1.392 (0.246)	4.595 (0.016*)	0.469 (0.629)
Adipsin (μg/mL)	5.9 (5.3/6.8)	6.4 (5.8/7.3)	6.7 (5.4/7.7)	6.1 (5.4/7.0)	0.195 (0.663)	0.229 (0.797)	0.870 (0.432)
Adiponectin (μg/mL)	33.2 (27.9/60.7)	35.7 (22.6/60.5)	47.2 (28.4/90.4)	50.4 (26.7/76.9)	2.404 (0.130)	1.492 (0.241)	1.885 (0.169)
Lipocalin 2 (μg/mL)	91.1 (75.2/103.2)	102.9 (83.6/115.0)	124.4 (101.8/140.9)	89.8 (71.0/109.7)	1.391 (0.245)	0.417 (0.661)	5.410 (0.008**)
PAI1 (ng/mL)^c^	20.9 (18.8/43.9)	29.3 (20.6/41.2)	35.4 (28.1/54.1)	39.3 (25.8/59.5)	3.434 (0.073)	0.402 (0.672)	0.613 (0.548)
Resistin (ng/mL)	24.3 (20.4/26.9)	23.9 (18.3/28.6)	24.2 (20.2/34.2)	22.5 (17.4/31.3)	0.543 (0.465)	0.700 (0.504)	1.987 (0.154)
MCP1 (pg/mL)	254.4 (203.8/417.9)	270.2 (224.2/374.1)	289.4 (250.8/446.7)	319.0 (275.5/348.5)	1.786 (0.190)	1.042 (0.365)	0.405 (0.670)
TNFα (pg/mL)	26.0 (13.6/42.2)	25.5 (22.0/43.2)	42.7 (29.6/57.4)	41.7 (34.5/54.3)	2.853 (0.100)	0.353 (0.705)	0.059 (0.943)
IL1-RA (pg/ml	254.6 (74.1/505.7)	221.9 (112.6/473.4)	316.4 (243.4/469.8)	344.6 (272.2/505.5)	5.479 (0.025*)	0.509 (0.606)	1.527 (0.231)
IL-10 (pg/mL)	6.1 (4.8/7.3)	5.5 (3.5/7.3)	6.0 (4.9/8.4)	5.6 (4.9/7.8)	0.398 (0.532)	1.049 (0.361)	1.803 (0.179)
IL-6 (pg/mL)^c^	0.8 (0.5/1.1)	0.8 (0.4/1.2)	0.8 (0.7/1.4)	0.7 (0.4/1.4)	0.087 (0.770)	0.959 (0.396)	0.226 (0.799)
BDNF (ng/mL)^c^	2.5 (0.6/6.9)^*¤*^	1.9 (1.0/3.9)	2.1 (0.4/5.9)	3.0 (1.2/6.4)	0.270 (0.607)	1.135 (0.334)	1.126 (0.336)

*IG* Intervention group (i.e., performing a structured resistance training intervention), *CG* Control group (i.e., receiving a less structured introduction to resistance training after the initial 15 weeks of unchanged low physical activity), *BL* Baseline, *6 m* Six months, *24-28 m* the period within the additional follow-up visit was set post-intervention/control period to enable more participants to participate. Fourteen analytes were within detectable limits, including: adiponectin, adipsin, brain-derived neurotrophic factor (BDNF), interleukin-1 receptor antagonist (IL1-RA), interleukin 6 (IL-6), interleukin 10 (IL-10), leptin, lipocalin-2, matrix metalloproteinase 2 (MMP2), matrix metalloproteinase 9 (MMP9), monocyte chemoattractant protein 1 (MCP1), plasminogen activator inhibitor 1 (PAI-1), resistin, and tumor necrosis factor α (TNF-α)

^§^Median (25th and 75th percentile); * *p* < 0.05; ** *p* < .001; ^¤^*n* = 17; ^¤¤^*n* = 16

^a^Linear Mixed Models (LMMs)

^b^Logaritmized values in the statistical analyses

^c^ + 10 was added as a constant before logaritmization to avoid negative values. The table mean/median values are the true values without the constant

There were no differences in how the IG and CG changed over time in BP and anthropometry, since there were no significant interactions between group and time for any of the variables (Table 4), regardless of whether women reporting MHT use at follow-ups were excluded or not (data not shown). VAT volume changed differently over time for the IG and CG, supported by a significant interaction between group and time (*p* = 0.041) (Table 4). However, at the 24–28 months follow-up, both groups showed increased VAT volumes compared to week 15, and the post hoc comparison did not show a significant difference between groups.

In the blood analyses, only ferritin and testosterone changed differently over time in the IG and CG, supported by significant interactions between group and time (*p* = 0.045 and 0.010, respectively) (Table 4). The results were consistent after excluding individuals reporting MHT use during follow-up (data not shown). At the 24–28 months visit, the IG had increased ferritin levels (from 80 to 85 µg/L) and decreased testosterone levels (from 0.59 to 0.53 nmol/L) compared to the week 15 visit. The post-hoc comparisons were not significantly different between groups at the 24–28 months visit (*p* = 0.210 and *p* = 0.408).

### Physical activity

**Fig. 3:**
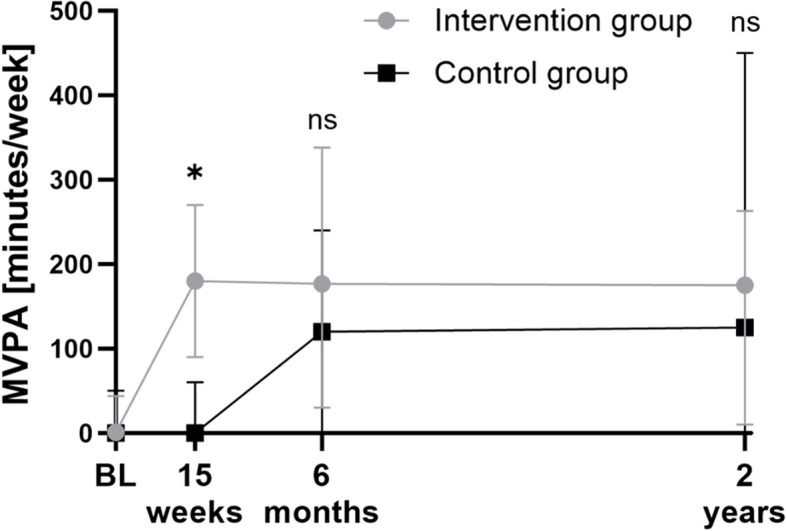
Moderate to vigorous physical activity (MVPA) across time. Moderate to vigorous physical activity (MVPA) minutes per week for the intervention group (i.e., performing a structured resistance training intervention) and control group (i.e., receiving a less structured introduction to resistance training after the initial 15 weeks of unchanged low physical activity). Data is expressed at all visits and telephone follow-ups in the RCT. The central tendency and dispersion are expressed as median and 25th/75th percentiles. Post-hoc between-group comparisons at each visit/telephone follow-up are performed with Bonferroni correction. * *p* < 0.01; ** *p* < 0.05; BL, baseline; ns, non significant

The MVPA is presented in Supplementary Table 1 and Fig. 3. All women increased their MVPA over time, regardless of group (*p* < 0.001) (Supplementary Table 1). There were significant differences in MVPA change over time between the groups, supported by a significant interaction between group and time (*p* = 0.006) (Supplementary Table 1). At the 6-month follow-up, the IG had increased their MVPA (from 1 to 177 min) while the CG had increased their MVPA (from 0 to 120 min) compared to the baseline in the RCT. Hence, the difference between the IG and the CG was not statistically significant (*p* = 0.944) and remained non-significant between groups at the 2-year follow-up (*p* = 0.578).

## Discussion

The purpose of the present study was to investigate the long-term effects of a 15-week RTI in postmenopausal women with VMS. As hypothesized, the RTI reduced MS-VMS frequency at least six months post-intervention compared to a CG receiving no structured intervention. However, over time, the intervention’s effect on MS-VMS frequency waned, as evidenced by decreased VMS frequency in both groups at the 2-year follow-up. Additionally, the initial HRQoL improvement in the IG after the RTI did not persist. While the cardiovascular risk markers ferritin, testosterone, and VAT volume exhibited statistically significant changes over time, we cannot confirm that the RTI improved the cardiovascular risk profile in the IG compared to the CG. There was not a clear trend in the change over time within either the IG or CG for these cardiovascular risk markers.

This is the first study with a 2-year follow-up after a resistance training intervention in postmenopausal women, with VMS frequency and HRQoL as the primary outcomes. A previous study has shown positive 4-year effects of aerobic exercise on VMS frequency and HFS [23], and a recent meta-analysis showed results in favor of PA as a potential non-pharmacological treatment for VMS [24]. However, the evidence supporting PA as VMS treatment remains insufficient [25]. While previous studies have compared the effect of aerobic [26, 27] or combined training [28] on VMS, studies comparing the effect of aerobic versus resistance training on VMS are lacking. Further research is needed to fill this gap in knowledge.

The improvements in HRQoL detected in the vasomotor, sleep, and menstrual domains of the WHQ immediately post-intervention in the IG were not maintained during follow-up, which are results supported by previous studies. In their systematic review and meta-analysis, Nguyen et al. could not show any evidence of exercise-mediated effects on general, social, and menopause-specific QoL scores [29]. In the present study, the lack of group-specific effects over time could be explained by several factors. The absolute values of the HRQoL domains at the 15-week visit could have already reached the “ceiling effect”, and thus be unlikely to show further improvement. In addition, as discussed in more detail later, both the IG and CG performed MVPA at a similar intensity during the follow-up period.

We have previously shown that the metabolism of atherogenic lipids, ferritin, biomarkers of inflammation, adipokines, sex hormones, and abdominal fat measured with MRI were affected by a 15-week RTI in healthy postmenopausal women [13, 14, 21]. In the present 2-year follow-up study, we cannot conclude that the RTI contributed to overall better cardiovascular health measured with surrogate markers compared to the CG, who received a free gym membership and an introductory session with a physiotherapist after their 15 weeks as controls. We observed group differences in VAT, ferritin, and testosterone changes over time. However, the clinical relevance can be questioned given the small absolute effect sizes and dramatically reduced effect of the intervention over time.

The plasma biomarkers and adipokines showed no uniform changes during the 2-year follow-up period. However, the significant time effect for the levels of MMP9 suggests a decrease over time regardless of group. Baseline differences between groups seen with lipocalin-2 and PAI-1 are probably a consequence of chance due to a reduction in the number of participants at the follow-up visit, as no baseline differences were observed in the larger RCT with greater power [14]. Different batches of the Luminex kits were used in the analyses, as they were run years apart, which can cause slight differences in absolute values. Sample degradation with long-term storage, transportation, and human error must also be considered.

Both the IG and CG increased in waist circumference over time, so we expected the proinflammatory biomarkers and adipokines would increase, considering that greater waist circumference is typically associated with a worsened inflammatory biomarker profile [30]. Therefore, the lack of uniform change in plasma biomarkers and adipokines could be considered a positive finding. Interestingly, most cardiovascular risk markers did not worsen significantly during follow-up, as would be expected over time for postmenopausal women.

The present follow-up study has several strengths. First and foremost, few PA intervention studies follow up on their participants prospectively for longer than one year [31], and long-term follow-ups of VMS and HRQoL outcomes after PA interventions in PM women are sparse. Secondly, this follow-up study includes all variables presented as outcomes in the original RCT to improve transparency and minimize publication bias. Thirdly, the data at each follow-up visit were collected and analyzed in the same manner as at the baseline and 15-week visits, enabling unbiased comparisons of the results across the visits. The chosen statistical method of analysis (LMMs) allows the use of all available data at each follow-up visit, which reduces the potential selection bias in the complete-case analysis and increases statistical power.

However, this study has several limitations. Firstly, the results must be interpreted in light of a non-blinded intervention where the primary outcomes were subjectively reported, which could increase the performance bias and therefore exaggerate the reduction in VMS frequency over time in the IG. Considering the observational study design, we cannot conclude about causal relationships. We cannot confirm that the maintained effects across time in VMS frequency were a consequence of the RTI per se and not changes in lifestyle or health-related habits. When conducting the original RCT, we intended to follow the participants after the RTI ended to observe if the effects on VMS frequency, HRQoL, and cardiovascular risk markers were maintained over time. Since the preliminary results in the original RCT pointed towards the health benefits of the performed RTI, we considered leaving the CG without PA encouragement to be unethical. However, it affects the possibility of finding differences between groups in the follow-up.

All women interested in participating in the original RCT were motivated to engage in PA from the beginning, which makes the results less transferable to the general population of postmenopausal women with VMS. The lack of more significant results could be explained by the fact that all women in both the IG and CG groups were engaged in MVPA after the initial 15-week RTI. The CG had significantly increased MVPA six months after the initial 15 weeks with low PA and kept that increased activity level to the 2-year follow-up. Ideally, we should have had a control group with low PA throughout the study, but we considered such a design unethical. However, the significantly different change over time in VMS frequency, with significant post-hoc comparisons between groups after six months, may indicate that the RTI had specific medium-term effects on VMS despite the same amount of self-reported PA during the follow-up.

The study also faced probable inadequacy in participant numbers, particularly evident in the secondary outcome analysis involving only 35 participants. The reduced statistical power resulting from participant drop-out may explain the lack of significant differences in VMS between IG and CG at the 2-year follow-up, even though natural cessation of VMS could have contributed to the observed results. The increased dropout rate was due to some participants starting MHT, while others chose not to continue participating. Notably, baseline characteristics showed no disparity between dropout and continuing participants, potentially mitigating the impact on generalizability despite the probable compromise in statistical power. In addition, since the study was developed from an RCT, the sampling, measures, and design are not optimized to fit the present research questions. Therefore, the results should be interpreted with caution but may still have clinical importance. Furthermore, simultaneous registration of diet could have been helpful to assess potentially changed dietary habits during the follow-up period, which could affect biomarkers. In addition, PA maintenance is self-reported in this study, lending insecurity and possibly overestimation of the amount of PA performed. However, this was the same for both the IG and CG groups so should not affect the comparisons between groups.

## Conclusions and clinical implications

A 15-week RTI could change MS-VMS frequency in the short and medium term. While the reduction in MS-VMS persisted in the IG, the CG gradually caught up with this effect throughout the study. After two years, there was no longer a difference compared with a group that received a less structured introduction to resistance training after 15 weeks of unchanged low physical activity. The RTI did not significantly improve HRQoL over time compared with a group that received a less structured introduction to resistance training, and neither did the RTI contribute to better preserved nor improved cardiovascular risk markers. Nevertheless, since we did not observe any significant worsening of the cardiovascular risk markers, as could be generally expected with time for postmenopausal women, the effectiveness of participating in a study promoting PA during the menopausal transition may still have had an impact on long-term health behavior.

The clinical implications from the results of this study may support the implementation of non-pharmacological alternatives to treat VMS with maintained results at least six months post-intervention. More research is needed to investigate the long-term effects of resistance training to improve or maintain cardiovascular health in postmenopausal women, explore other modes of PA, and focus on strategies to motivate postmenopausal women who could benefit from PA to engage in such training consistently over time.

## Supplementary Information

Supplementary Material 1.

## Data Availability

The datasets used and/or analyzed during the current study are available from the corresponding author upon reasonable request.

## Abbreviations

ASAT: Abdominal subcutaneous adipose tissue
BDNF: Brain-Derived Neurotrophic Factor
BMI: Body Mass Index
BP: Blood Pressure
CI: Confidence Interval
CG: Control Group
CRP: C-Reactive Protein
ELISA: Enzyme-Linked Immunosorbent Assay
HAW: Half Abdominal Width
HDL: High-Density Lipoprotein
HFS: Hot Flush Score
HRQoL: Health-related quality of life
IG: Intervention Group
IL-1RA: Interleukin-1 Receptor Antagonist
IL-10: Interleukin-10
IL-4: Interleukin-4
IL-6: Interleukin-6
IPAQ: International Physical Activity Questionnaire
LDL: Low-Density Lipoprotein
LMMs: Linear Mixed Models
MCP-1: Monocyte Chemoattractant Protein-1
MET: Metabolic equivalents of task
MMP2: Matrix Metalloproteinase 2
MMP9: Matrix Metalloproteinase 9
MRI: Magnetic Resonance Imaging
MS-VMS: Moderate to Severe Vasomotor Symptoms
MVPA: Moderate-to-Vigorous Physical Activity
PA: Physical activity
PAI-1: Plasminogen Activator Inhibitor-1
PCS: Physical Component Summary
RCT: Randomized Controlled Trial
RTI: Resistance training intervention
SAT: Subcutaneous adipose tissue
SAD: Sagittal abdominal diameter
SD: Standard Deviation
SF-36: Short Form Health Survey 36-items
SHBG: Sex Hormone-Binding Globulin
TAAT: Total Abdominal Adipose Tissue
TNF-α: Tumor Necrosis Factor-alpha
VAT: Visceral Adipose Tissue
VMET: Vigorous Metabolic Equivalent Task
WC: Waist Circumference
WHQ: Women’s Health Questionnaire
