# Supplementary material for: A 2-year follow-up to a randomized controlled trial on resistance training in postmenopausal women: vasomotor symptoms, quality of life and cardiovascular risk markers
Source: BMC Womens Health. 2024 Sep 13;24:511. doi: 10.1186/s12905-024-03351-1 (PMC11396825; doi:10.1186/s12905-024-03351-1)
Supplement: Supplementary file 1 — Supplementary Material 1. [file 12905_2024_3351_MOESM1_ESM.docx]

Supplementary table 1. Moderate to vigorous physical activity (MVPA)

| Variables | IG (n = 28) | | | CG (n = 29) | | | Type III fixed effects^a^ | | |
| --- | --- | --- | --- | --- | --- | --- | --- | --- | --- |
|  | **BL**  **n = 28** | **6m**  **n = 26** | **2y**  **n = 24** | **BL**  **n = 29** | **6m**  **n = 27** | **2y** | **Group F(p-value)** | **Time F(p-value)** | **Group x Time  F(p-value)** |
| **IPAQ questionnaire**^§,b^ |  |  |  |  |  | **n = 23** |  |  |  |
| MVPA (minutes/week) | 1 (0/44) | 177 (30/338) | 175 (10/263) | 0 (0/50) | 120 (0/240) | 125 (0/450) | 2.92 (0.950) | 11.97 (<0.001) | 4.52 (0.006) |
| IG, Intervention group (i.e., performing a structured resistance training intervention); CG, Control group (i.e., receiving a less structured introduction to resistance training after the initial 15 weeks of unchanged low physical activity); BL, baseline; IPAQ, international physical activity questionnaire; MVPA, moderate to vigorous physical activity; ^§^ Median (25^th^ /75^th^ percentile);  ^a^ Linear Mixed Models (LMMs); ^b^ Logaritmized values in the statistical analyses. | | | | | | | | | |

Supplementary table 2. Comparison of baseline characteristics, lost to follow-up vs. follow-up participants

| Variables | Lost to follow-up (n = 30) | Follow-up (n = 35) | Baseline comparison |
| --- | --- | --- | --- |
|  | **n = 30** | **n = 35** | **P-value** |
| Age (years) ^~,1^ | 55 ± 6 | 56 ± 5 | 0.583 |
| Menopausal time ^§,2^ | 32 (14/82)^¤^ | 41 (21/77)^¤^ | 0.497 |
| **Clinical information** | **n = 30** | **n = 35** |  |
| SBP (mmHg) ^~,1^ | 127 ± 14 | 132 ± 16 | 0.181 |
| DBP (mmHg) ^~,1^ | 78 ± 9 | 79 ± 9 | 0.527 |
| Weight (kg) ^~,1^ | 74.4 ± 10.9 | 75.0 ± 12.3 | 0.831 |
| BMI (kg/m^2^) ^~,1^ | 27.5 ± 3.7 | 27.6 ± 3.9 | 0.921 |
| WC (cm) ^~,1^ | 90.5 ± 13.3 | 90.3 ± 13.5 | 0.943 |
| HAW (cm) ^~,1^ | 15.8 ± 2.0 | 18.2 ± 2.6 | **<0.001**** |
| SAD (cm) ^~,1^ | 22.5 ± 2.5 | 21.7 ± 2.9 | 0.251 |
| **MRI** | **n = 19** | **n = 28** |  |
| ASAT (L)?) ^§,2^ | 8.0 (5.3/9.0) | 8.3 (7.1/11.9) | 0.326 |
| VAT (L) ^§,2^ | 1.8 (1.5/4.3) | 2.6 (1.7/3.7)^##^ | 0.326 |
| VAT-ratio (%) ^§,2^ | 21.4 (17.7/28.9) | 23.0 (17.5/26.7)^##^ | 0.606 |
| **Blood analyses** | **n = 11^!^** | **n = 35** |  |
| LDL (mmol/L) ^~,1^ | 3.3 ± 0.7 | 3.6 ± 1.0 | 0.419 |
| HDL (mmol/L) ^~,1^ | 1.9 ± 0.6 | 2.1 ± 0.8 | 0.629 |
| ApoA1 (g/L) ^~,1^ | 1.7 ± 0.3^¤¤¤^ | 1.8 ± 0.3 | 0.465 |
| ApoB (g/L) ^~,1^ | 1.0 ± 0.2^¤¤¤^ | 1.1 ± 0.3^#^ | 0.437 |
| Ferritin (µg/L) ^§,2^ | 68 (50/94)^¤¤¤^ | 128 (61/160) | 0.244 |
| SBP, Systolic blood pressure; DBP, Diastolic blood pressure; BMI, Body Mass Index; WC, Waist circumference; HAW, Half abdominal width; SAD, Sagittal abdominal diameter; ASAT, Abdominal subcutaneous adipose tissue; VAT, Visceral adipose tissue; VAT-ratio, ratio in percent to total body adipose tissue; LDL, Low-dense lipoprotein; HDL, High-dense lipoprotein; ApoA1, Apolipoprotein A1; ApoB, Apolipoprotein B; hsCRP, High sensitive C-reactive protein; SHBG, Sex Hormone Binding Globulin; MRI, magnetic resonance imaging; ^1^ Independent samples T-test, ^2^ Mann-Whitney U-test, ~ Mean ± SD, ^§^ Median (25^th^ and 75^th^ percentile), * p < 0.05, ** p<0.01, ¤ n = 24, ¤¤ n = 28, ¤¤¤ n = 7, # n = 34, ## n = 27. | | | |
